# Accuracy of matrix-assisted LASER desorption ionization–time of flight mass spectrometry for identification of *Candida*

**DOI:** 10.1042/BSR20190859

**Published:** 2019-10-11

**Authors:** Tian-Ao Xie, Ye-Ling Liu, Chuan Liang, Yuan-Yuan Huang, Jin-Wei Li, Zhong-Wei Li, Shu-Jin Fan, Jin-Tao Chen, Yong Xia, Xiao-Yan Li, Shi Ouyang, Tian-Xing Ji, Xu-Guang Guo

**Affiliations:** 1 Department of Clinical Medicine, The Third Clinical School of Guangzhou Medical University, Guangzhou 511436, China; 2 Department of Clinical Laboratory Medicine, The Third Affiliated Hospital of Guangzhou Medical University, Guangzhou 510150, China; 3 Key Laboratory for Major Obstetric Diseases of Guangdong Province, Guangzhou 510150, China; 4 Key Laboratory of Reproduction and Genetics of Guangdong Higher Education Institutes, Guangzhou 510150, China; 5 Department of Laboratory Medicine, The Affiliated Shunde Hospital of Guangzhou Medical University, Foshan, China; 6 Department of Infectious Disease, The Fifth Affiliated Hospital of Guangzhou Medical University, Guangzhou 510000, China; 7 Department of Clinical Medicine, The Second Affiliated Hospital of Guangzhou Medical University, Guangzhou 511436, China

**Keywords:** Candidemia, diagnosis, MALDI-TOF-MS, Meta-analysis

## Abstract

**Background**: *Candida* is a fungus that causes various types of candidemia, which is the fourth major infectious disease of the blood system. MALDI-TOF-MS is a simple and rapid detection instrument. The aim of the present study was to verify the accuracy of MALDI-TOF-MS in detecting *Candida*.

**Method**: A pooled analysis of articles on MALDI-TOF-MS for diagnosis of candidemia was performed. The quality of original research was assessed using the Quality Assessment of Diagnostic Accuracy Studies (QUADAS-2) guidelines. Stata 12.0 software was used to merge the correct identification rates of *Candida* and *Candida* subspecies and obtain pooled sensitivity and specificity of the diagnostic methods. Heterogeneity was found in the subgroup analysis of the included articles. Hence, we explored the factors causing the heterogeneity and its impact on the overall situation. Sensitivity analysis was used to examine the effect of *Candida* level on total response. Egger’s test was used to evaluate the publication bias of the included articles.

**Results**: A total of 16 articles in Pubmed, 79 articles in Embase, 1 article in Cochrane Library, 30 articles in Web of Science and 3 from other sources were identified, of which 10 articles were included based on the inclusion and exclusion criteria. The overall identification accuracy was 100%.

**Conclusion:** The accuracy of MALDI-TOF-MS for the identification of *Candida* was 100%. Further research is necessary to determine whether MALDI-TOF-MS can be used as a clinical diagnostic standard for the identification of *Candida*.

## Background

*Candida* is a common parasitic fungus in human respiratory, gastrointestinal and urogenital systems. It can cause candidemia by invading the tissues, and even death when human immunity decreases [1]. Candidemia is a blood infection with increased morbidity and mortality rate of up to 40% [2]. The prognosis and mortality of patients are related to the distribution of *Candida* species. Candidemia is the fourth common cause of death among infectious diseases of the blood system [3]. At present, among all types of *Candida* infections, *Candida albicans* is predominant, but the proportion of non-*C. albicans* is rising [4]. *Candida glabrata* and *Candida krusei* are less susceptible to common azole anti-fungal drugs and can develop natural resistance, which may be related to the increasing incidence of candidemia [1]. Early diagnosis is the key to successful treatment of candidemia [5]. *Candida* can be examined in many ways, such as direct examination, Gram staining, culture etc. But these checks are time-consuming and sometimes the results may be uncertain, which delays patient’s treatment time and aggravates his/her condition [5]. A simpler, faster and higher accuracy-test method is urgently required for clinical examination.

MALDI-TOF-MS is a new type of soft ionization mass spectrometry, with its own spectral database [6], which was introduced as a rapid method for identifying bacteria and yeast [7]. It is a powerful device for proteomic analysis [8]. Its advantages are simple and intuitive [9]. It can be used to directly identify macromolecule mixtures, without the need to separate and slice [10]. The species can be identified in three steps: first, the sample collected is placed on a specially designed metal target plate. Then the instrument conducts the measurements. Finally, the model is used to infer the species by combining spectra with well-known and/or well-defined species of spectral databases [11]. Moreover, it has high sensitivity, rapid detection [12], high throughput, large-scale identification of proteins and determination of the molecular weight of biological macromolecules [13]. Although the efficiency of MALDI-TOF-MS is well established, its accuracy needs to be urgently tested, in order to facilitate its use in clinical examination. There is no meta-analysis on the diagnostic accuracy of MALDI-TOF-MS for candidemia in evidence-based medicine. The aim of the present study was to evaluate the accuracy of MALDI-TOF-MS in the identification of *Candida* species, in order to provide a new means and the gold standard for clinical diagnosis.

## Materials and methods

### Study design

Our study date is from December 2017 to date. A systematic review of the diagnostic accuracy of MALDI-TOF-MS in candidemia was performed, followed by a meta-analysis.

### Search strategy

We searched the keywords ‘MALDI-TOF-MS’ and ‘Candidemia’ among four databases of Pubmed, Embase, Cochrane Library and Web of Science, and collected the articles published before February 2018. Four investigators (T.-A. Xie, Y.L. Liu, Y.Y. Huang, and C. Liang) independently screened the retrieved publications according to pre-established inclusion and exclusion criteria and extracted the data from the papers. Any differences were resolved by discussing with another researcher (J.-W. Li).

### Inclusion criteria and data extraction

Before reviewing the articles, the researchers established the criteria for inclusion and exclusion. The inclusion criteria were formulated based on the PICOS criteria. (1) Objectives: Clinical specimens were identified as *Candida* or standard strains by reference methods. (2) Types of study: test of diagnostic accuracy, the data of identifying *Candida* species level can be extracted, limited to English language. (3) Measurement indicators: accuracy. (4) Diagnostic experimental methods: the identification of *Candida* by MALDI-TOF-MS. The following studies were excluded: duplicate studies, abstracts, conference summaries, case reports/reviews/posters; lack of a reference method or a detailed number of isolates. According to the category of the strain and the MALDI-TOF-MS system, the number of isolates was correctly identified and total isolates were abstracted. After preliminary screening of the documents that met the inclusion criteria, we used EndNoteX8 software for document management and extracted data from the articles, including the author name, year of publication, study design, strain distribution area, detection instrument, detection system, and strain source, in an Excel spreadsheet.

### Quality assessment

The quality of the included studies was assessed using the Quality Assessment of Diagnostic Accuracy Studies (QUADAS-2) guidelines [14]. The guidelines comprise four key domains that judge bias and applicability of the studies by reviewing how the patients were selected, index test, reference standard, and the flow of patients through the study. These variables were entered in a main data Excel spreadsheet.

### Data synthesis and analysis

The main measurement index was the correct identification rate of *Candida*. The correct identification rate is the ratio of the number of strains identified by MALDI-TOF-MS to the number of strains identified by the reference methods in the study. Stata 12.0 software was used to merge the correct identification rates of *Candida* and *Candida* subspecies. *I^2^* measure was used to estimate heterogeneity between studies. In the case of greater heterogeneity, subgroup analysis of the included studies was conducted to explore the impact of factors causing heterogeneity on the overall effect and the degree of impact [15]. Sensitivity analysis was used to study how the effect of *Candida* level data could be apportioned to the total response [16]. Egger’s test was used to evaluate the publication bias of the included studies [17]. All analyses were performed with Stata statistical software package, version 12.0 (Stata Corp LP, College Station, U.S.A.).

## Results

### Eligible studies

**Figure 1:**
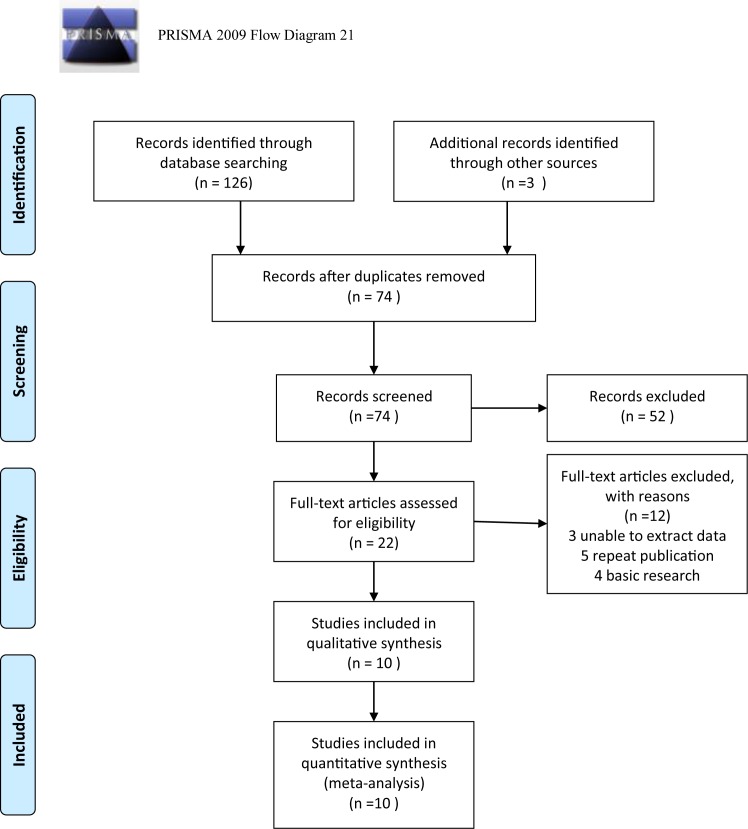
Flow diagram for systematic article search *From:* Moher D., Liberati A., Tetzlaff J., Altman D.G., The PRISMA Group (2009) *P*referred *R*eporting *I*tems for *S*ystematic Reviews and *M*eta-Analyses: the PRISMA Statement. *PLoS Med.***6**(6), e1000097. doi:10.1371/journal.pmed1000097; **for more information, visit www.prisma-statement.org.**

After a comprehensive database search, we identified 16 articles in Pubmed, 79 in Embase, 1 in Cochrane Library, 30 in Web of Science and 3 from other sources. Of the 129 references, 55 were duplicate. After reviewing the title and abstract, a total of 22 articles remained for full-text screening. Of these, two articles were excluded due to inability to extract data; five articles were discarded as duplicates, and four basic research articles were excluded. Finally, ten articles [1,2,5,7,18–23] were included and their research data were extracted for meta-analysis (Figure 1).

### Data presented in the studies

We extracted information such as the author name, year of publication, study design, strain distribution area, detection instrument, detection system, strain source etc., from the included studies (Table 1). The identification accuracy rate of species of these studies is summarized in Table 2.

**Table 1:** Characteristics of included articles

Authors	Year	Study design	Geographical distribution of strains	System	System database	Source of strains	Ref. method(s)	Events	Total	Correct rate
Stevenson [18]	2010	Retrospective	America	Bruker	A spectral database library with m/z ratios of 2000– 20000 Da for 109 types	194 clinical isolates	Sequencing	192	194	99%
Yaman [5]	2012	Retrospective	Turkey	Bruker	The FlexAnalysis software version 3.0, the MALDI Biotyper software version 2.0	281 clinical isolates	Sequencing	281	281	100%
Lavergne [19]	2013	Retrospective	France	bioMérieux	The spectral database MS-ID version 1	66 clinical and reference strains	Routine laboratory technique*	64	66	97%
Pulcrano [7]	2013	Retrospective	Italy	Bruker	Self-established database	82 clinical isolates	Sequencing	82	82	100%
Taj-Aldeen [1]	2014	Retrospective	Netherlands	Bruker	Biotyper 3.0 system	201 clinical isolates	Sequencing	201	201	100%
Andersen [20]	2016	Retrospective	Norway	Bruker	Biotyper 3.1, Maldi Biotyper Compass version 4.1	183 clinical isolates	Sequencing	183	183	100%
Chapman [2]	2017	Prospective	Australia	Bruker	Biotyper database v3.1	Nationwide active laboratory-based surveillance for candidemia over 1 year (within 2014–2015)	Sequencing	485	548	89%
Trouvé [21]	2017	Prospective	Belgium	Bruker	Microflex LT Biotyper	355 clinical isolates	Sequencing	355	355	100%
Wu [22]	2017	Retrospective	Taiwan	Bruker	The IBM Statistical Package for the Social Sciences, version 18.0	270 clinical isolates	Sequencing	270	270	100%
Li [23]	2018	Retrospective	Taiwan	bioMérieux	The MS-ID version 2.0, the IVD Database	512 clinical isolates	Sequencing	494	510	97%

Abbreviation: IVD, *in vitro* device.

* : *C. albicans* was identified by CHROMagar chromogenic medium (Becton Dickinson, Heidebelberg, Germany); *C. glabrata, C. tropicalis, C. parapsilosis* were identified by ID32 C (bioMérieux, la Balme, France).

**Table 2:** The identification accuracy rate of species from included articles

Study	Year	Species	Events	Total	Correct rate
Stevenson [18]	2010	*C. albicans*	20	20	100%
		*C. glabrata*	11	11	100%
		*C. tropicalis*	8	8	100%
		*C. parapsilosis*	17	17	100%
		*C. catenulata*	2	2	100%
		*C. dubliniensis*	12	12	100%
		*C. guilliermondii*	15	15	100%
		*C. haemulonii*	2	2	100%
		*C. kefyr*	10	10	100%
		*C. lipolytica*	9	9	100%
		*C. lusitania*	10	10	100%
		*C. metapsilosis*	8	8	100%
		*C. orthopsilosis*	21	21	100%
		*C. pelliculosa*	10	10	100%
		*C. rugosa*	6	7	86%
Yaman [5]	2012	*C. albicans*	174	174	100%
		*C. glabrata*	25	25	100%
		*C. tropicalis*	42	42	100%
		*C. parapsilosis*	19	19	100%
		*C. dubliniensis*	1	1	100%
		*C. kefyr*	5	5	100%
		*C. krusei*	10	10	100%
		*C. lambica*	1	1	100%
		*C. lusitaniae*	4	4	100%
Lavergne [19]	2013	*C. albicans*	21	21	100%
		*C. glabrata*	11	12	92%
		*C. tropicalis*	3	4	75%
		*C. parapsilosis*	5	5	100%
		*C. dubliniensis*	4	4	100%
		*C. guilliermondii*	6	6	100%
		*C. inconspicua*	3	3	100%
		*C. kefyr*	3	3	100%
		*C. krusei*	5	5	100%
		*C. lusitaniae*	3	3	100%
Pulcrano [7]	2013	*C. glabrata*	11	11	100%
		*C. tropicalis*	3	4	75%
		*C. parapsilosis*	5	5	100%
		*C. guillermondii*	6	6	100%
		*C. krusei*	1	1	100%
		C. lipolytica	1	1	100%
		L. elongisporus*	1	1	100%
Taj-Aldeen [1]	2014	*C. albicans*	68	68	100%
		*C. glabrata*	38	38	100%
		*C. tropicalis*	36	36	100%
		*C. parapsilosis*	34	34	100%
		*C. dubliniensis*	3	3	100%
		*C. intermedia*	1	1	100%
		*C. orthopsilosis*	8	8	100%
		*C. pararugosa*	2	2	100%
		L. elongisporus*	1	1	100%
Andersen [20]	2016	*C. glabrata*	183	183	100%
Trouvé [21]	2017	*C. albicans*	179	179	100%
		*C. glabrata*	97	97	100%
		*C. tropicalis*	20	20	100%
		*C. parapsilosis*	35	35	100%
		*C. dubliniensis*	4	4	100%
		*C. guilliermondii*	9	9	100%
		*C. krusei*	4	4	100%
		*C. lusitaniae*	4	4	100%
		*C. fermentati*	2	2	100%
		*C. palmioleophila*	1	1	100%
Wu [22]	2017	*C. albicans*	116	116	100%
		*C. glabrata*	27	27	100%
		*C. tropicalis*	47	47	100%
		*C. parapsilosis*	61	61	100%
		other *Candida*	19	19	100%
Li [23]	2018	*C. albicans*	249	253	98%
		*C. glabrata*	60	60	100%
		*C. tropicalis*	107	110	97%
		*C. parapsilosis*	56	60	93%
		*C. dubliniensis*	2	3	67%
		*C. guilliermondi*	8	8	100%
		*C. haemulonii*	4	4	100%
		*C. krusei*	5	5	100%
		*C. nivariensis*	0	2	0%
		*C. pelliculosa*	1	1	100%
		*C. rugosa*	1	1	100%

* : *Lodderomyces elongisporus*.

### QUADAS-2 results of meta-analyzed publications

The subjects in the ten studies were identified by conventional methods or genetic analysis before validation. The quality evaluation of the included articles was conducted (Table 3).

**Table 3:** The quality evaluation results for each study included in the meta-analysis

Study	Year	QUADAS-2										
		1	2	3	4	5	6	7	8	9	10	11
Stevenson [18]	2010	Y	N	Y	N	UC	Y	Y	Y	N	Y	N
Yaman [5]	2012	Y	N	Y	N	Y	Y	N	Y	N	Y	Y
Lavergne [19]	2013	Y	N	Y	N	UC	UC	Y	Y	Y	Y	Y
Pulcrano [7]	2013	Y	N	Y	Y	Y	Y	N	Y	N	N	Y
Taj-Aldeen [1]	2014	Y	N	Y	N	Y	Y	Y	Y	Y	Y	Y
Andersen [20]	2016	Y	N	N	N	UC	Y	Y	Y	Y	Y	Y
Chapman [2]	2017	Y	N	N	N	UC	Y	Y	Y	Y	Y	Y
Trouvé [21]	2017	Y	N	Y	N	UC	Y	Y	Y	Y	Y	N
Wu [22]	2017	Y	N	Y	N	UC	Y	Y	Y	Y	Y	N
Li [23]	2018	Y	N	Y	N	Y	Y	N	Y	Y	Y	N

Abbreviations: N, No; UC, Unclear; Y, Yes.

### Overall meta-analysis

**Figure 2:**
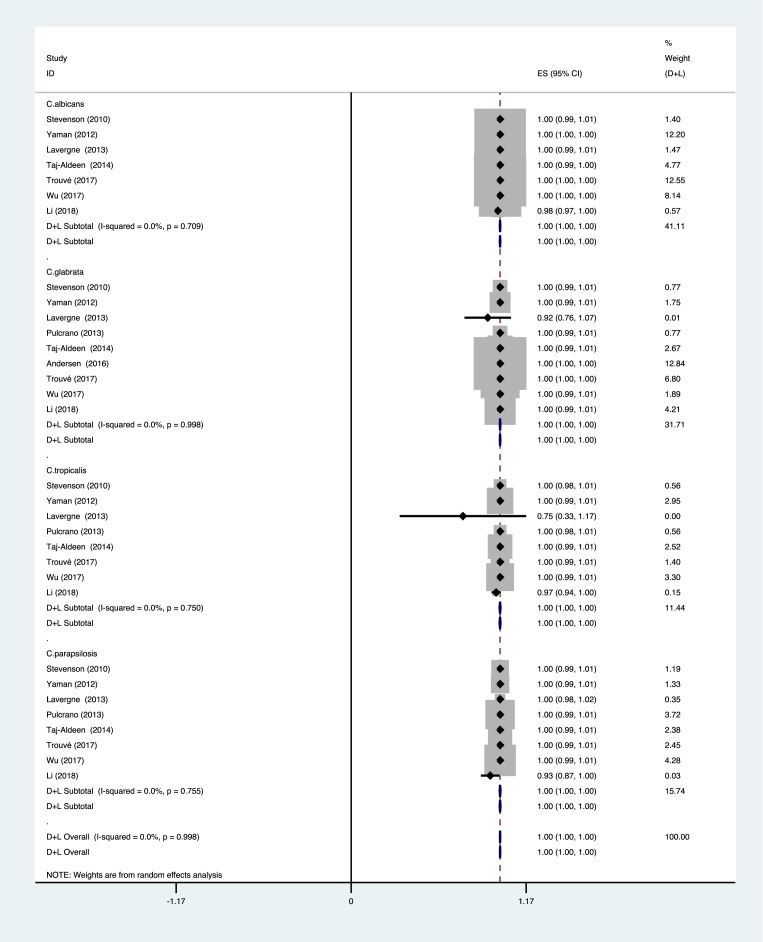
Forest plot for the meta-analysis of the *Candida* identification ratio at the genus level

In the ten included articles, a total of 1854 *Candida* isolates were assessed. The overall statistical results of the meta-analysis at the genus level identification were summarized by forest plots of the random-effects model using Stata 12.0 software (Figure 2). Identification accuracy of *C. albicans* was at 100% (*P*=0.709; *I^2^* = 0.0%), *C. glabrata* was at 100% (*P*=0.998; *I^2^* = 0.0%), *C. tropicalis* was at 100% (*P*=0.750; *I^2^* = 0.0%), and *C. parapsilosis* was at 100% (*P*=0.755; *I^2^* = 0.0%). The overall identification accuracy was at 100% (*P*=0.998; *I^2^* = 0.0%).

### Subgroup meta-analyses

**Figure 3:**
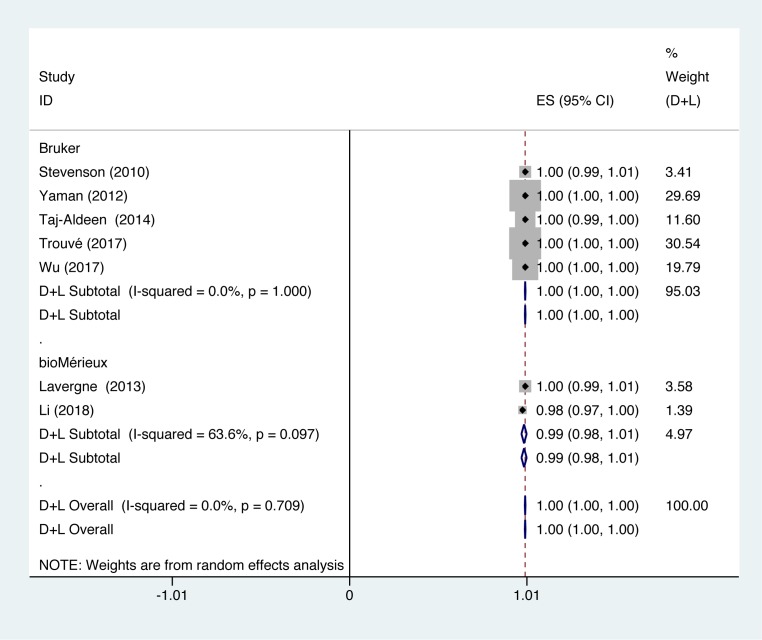
Forest plot for the subgroup analysis of the *C. albicans* identification ratio on the instrumental aspects

Subgroup analysis was performed on the instruments of the collected data. We combined and compared different instruments (Bruker; bioMérieux). In studies using Bruker to identify *C. albicans*, identification accuracy of *C. albicans* was at 100% (*P*=1.000; *I^2^* = 0.0%). In studies using bioMérieux to identify *C. albicans*, identification accuracy of *C. albicans* was at 99% (*P*=0.097; *I^2^* = 63.6%) (Figure 3).

**Figure 4:**
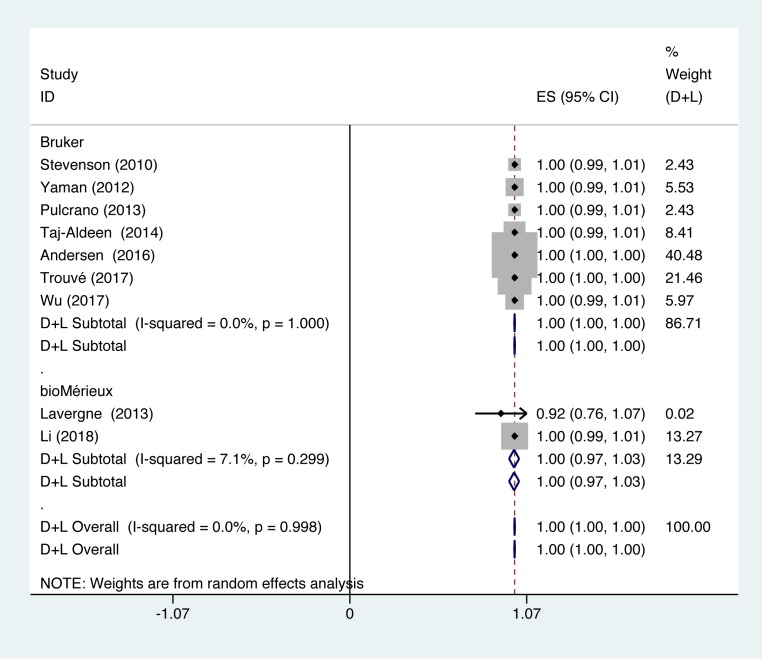
Forest plot for the subgroup analysis of the *C. glabrata* identification ratio on the instrumental aspects

In studies using Bruker to identify *C. glabrata*, identification accuracy of *C. glabrata* was at 100% (*P*=1.000; *I^2^* = 0.0%). In studies using bioMérieux to identify *C. glabrata*, identification accuracy of *C. glabrata* was at 100% (*P*=0.299; *I^2^* = 7.1%) (Figure 4).

**Figure 5:**
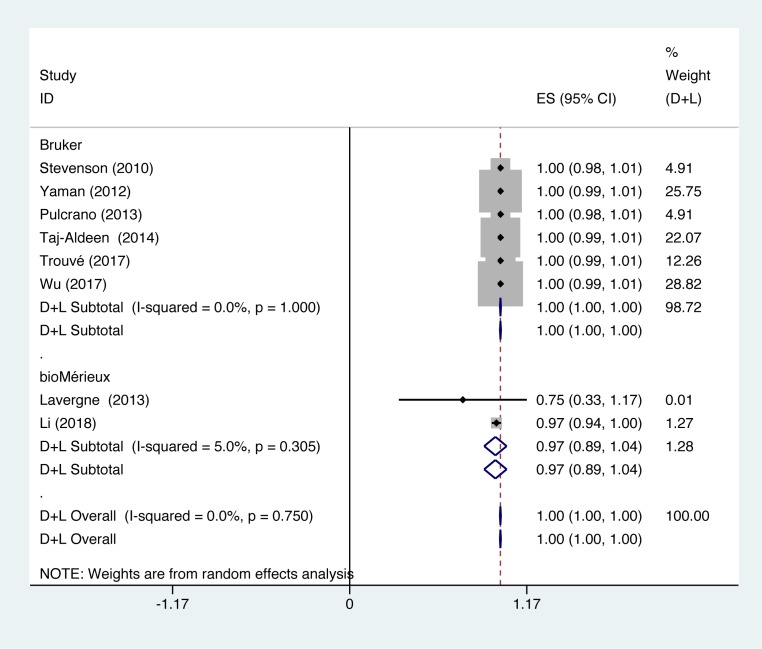
Forest plot for the subgroup analysis of the *C. tropicalis* identification ratio on the instrumental aspects

In studies using Bruker to identify *C. tropicalis*, identification accuracy of *C. tropicalis* was at 100% (*P*=1.000; *I^2^* = 0.0%). In studies using bioMérieux to identify *C. tropicalis*, identification accuracy of *C. tropicalis* was at 100% (*P*=0.305; *I^2^* = 5.0%) (Figure 5).

**Figure 6:**
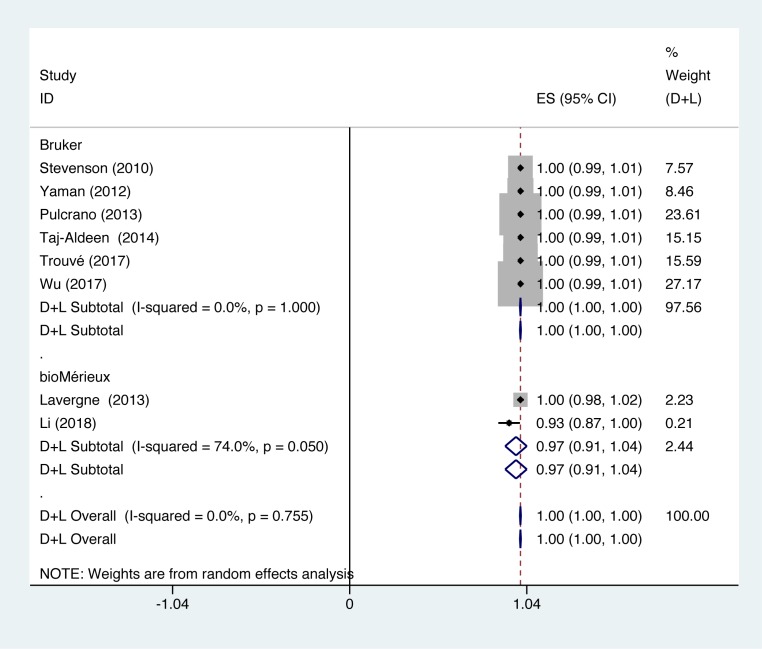
Forest plot for the subgroup analysis of the *C. parapsilosis* identification ratio on the instrumental aspects

In studies using Bruker to identify *C. parapsilosis*, identification accuracy of *C. parapsilosis* was at 100% (*P*=1.000; *I^2^* = 0.0%). In studies using bioMérieux to identify *C. parapsilosis*, identification accuracy of *C. parapsilosis* was at 100% (*P*=0.050; *I^2^* = 74.0%) (Figure 6).

**Figure 7:**
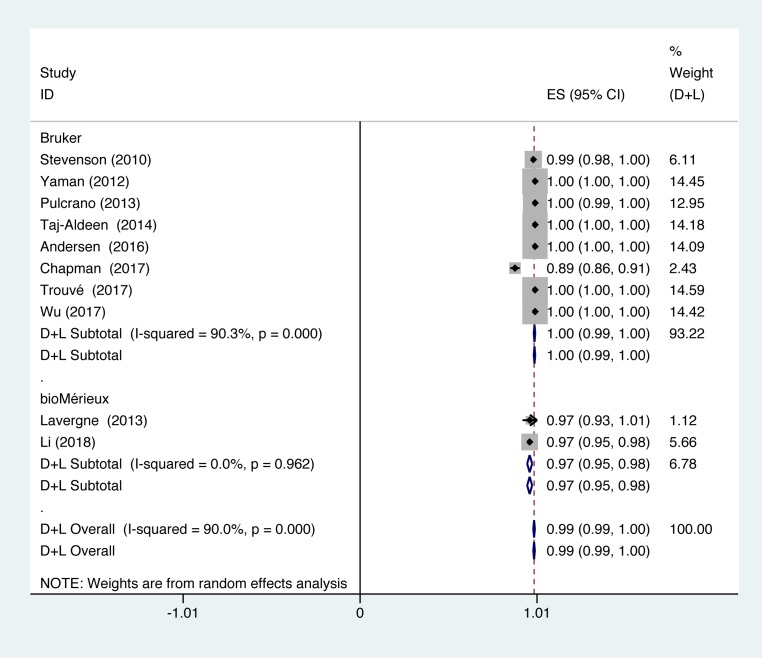
Forest plot for the subgroup analysis of the system

In studies using Bruker to identify *Candida*, identification accuracy of *Candida* was at 100% (*P*=0.000; *I^2^* = 90.3%). In studies using bioMérieux to identify *Candida*, identification accuracy of *Candida* was at 97% (*P*=0.962; *I^2^* = 0.0%) (Figure 7).

### Sensitivity analysis

**Figure 8:**
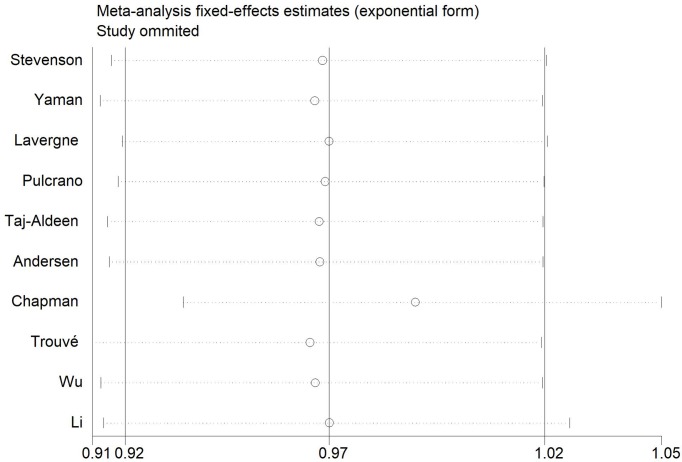
Sensitivity analysis of *Candida* levels

The influence of the included articles on the total pooled efficacy was investigated using the sensitivity analysis. The sensitivity analysis refers to a new meta-analysis conducted every time a certain study is deleted, in which the combined effect is compared with the overall effect to detect any change in the results. The vertical solid line of 0.97 in the middle represents the overall combined effect. The left and right vertical solid lines represent the upper and lower limits of the 95% confidence interval of the total pooled effect (Figure 8).

### Assessment of publication bias

**Figure 9:**
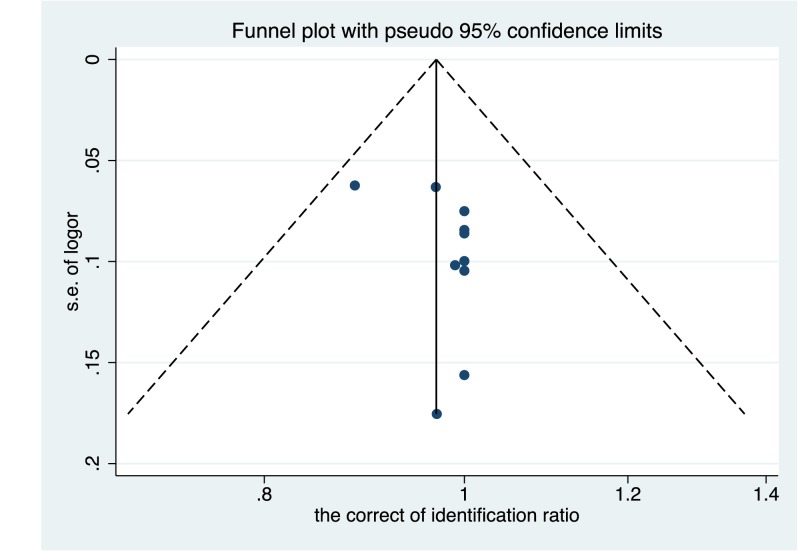
The funnel plot of the combined *Candida*

Statistically significant results are more likely to be accepted and published in similar studies than non-statistically significant studies. The control of publication bias is difficult and influences the results of systematic evaluation. The *P*-values of the funnel chart and Egger’s test were used for the evaluation of publication bias in the present study. The combined results of *Candida* (t = −2.04; *P*=0.076) are shown in Figure 9.

## Discussion

In the present study, we focused on the importance of rapid identification of *Candida* by MALDI-TOF-MS. The accuracy of the MALDI-TOF MS for clinical *Candida* isolates identified by the gold standard method was considered to be in accordance with the conditions of the meta-analysis. The results illustrated that the overall identification accuracy was 100% (*P*=0.998; *I^2^* = 0.0%), indicating that MALDI-TOF-MS is very accurate in identifying *Candida*.

In our study, we mainly investigated four kinds of common clinical *Candida* species (*C. albicans, C. glabrata, C. tropicalis, C. parapsilosis*). Due to the more frequent use of azole anti-fungals and invasive procedures, other *Candida* species have been increasing [24]. In the articles we extracted, the accuracy of identification of most of the rare yeasts was very high. For example, *C. guilliermond*i (100%), *C. krusei* (100%), *C. dubliniensis* (100%), and so on. But there were a few exceptions, like *C. dubliniensis* (2/3) [23] and *C. rugosa* (6/7) [18]. Because of too few samples, in order to ensure scientific preciseness and avoid excessive heterogeneity, we cannot verify the accuracy of MALDI-TOF-MS in these rare *Candida* species.

In subgroup analysis, we compared the accuracy of two MALDI-TOF-MS systems in identifying *Candida* species. Eight of the ten articles were Bruker MALDI Biotyper system, and 2114 strains were studied. The accuracy of the Bruker MALDI Biotyper System in identifying Candida was 100%. Two were bioMérieuxVitek MS system, and 576 strains were studied. The accuracy of bioMérieuxVitek MS system in identifying *Candida* was 97%.

The results of instrument subgroup analysis of collected data indicated differences in identification capabilities of the two commercial MALDI-TOF-MS systems (Bruker MALDI Biotyper and bioMérieuxVitek MS). According to the results of bioMérieuxVitek MS detection of different subspecies of *Candida*, the overall heterogeneity of *Candida* was higher than that of Bruker MALDI Biotyper, especially for *C. albicans* and *C. parapsilosis*. The *I^2^* values of *C. albicans* and *C. parapsilosis* detected by bioMérieuxVitek MS were 63.6 and 74.0%, respectively, suggesting moderate heterogeneity. However, the *I^2^* of different subspecies detected by Bruker MALDI Biotyper was zero, indicating no heterogeneity.

Two of the ten included articles used the bioMérieux Vitek MS system. Analysis of these two articles revealed several factors that can explain the observed heterogeneity. We found that Li et al. [23] mentioned that several isolates were identified as ‘bad spectrum during acquisition’ in the discussion of the article. We think this may affect the results of mass spectrometry to some extent. Besides, the database of VITEK MS used in the present study was*in vitro* devices (IVD), not the VITEK MS database for research only (RUO), which would lead to discrepant results. In addition, many factors, such as the difference between *Candida* spp., the reference method, were incorrectly identified, the protein profiles of *Candida* collected in the database were incomplete, the strains used in the study were not identified by a unified gold standard, the ease of polymicrobial bloodstream infections when directly identifying the *Candida* from blood culture, manmade operation level etc., may lead to heterogeneity, thus reducing the efficiency of the study.

The results of the present study illustrated that the level of *Candida* was distributed on both sides of the axis (0.97) and did not exceed the 95% confidence interval (0.92–1.02). No single study result affected the total combined effect. In Egger’s test, *P*>0.1 indicates no publication bias. The results of the present study revealed *P*=0.076 suggesting minor publication bias, which is permissible in a meta-analysis.

Although classical molecular identification methods continue to be used in clinical diagnosis, MALDI-TOF-MS is increasingly used in clinical microbiology laboratories and has become a gold standard [25,26]. The MALDI-TOF test procedure was completed in approximately 13 min, while the conventional identification method required 24–48 h [5]. According to the data collected in this study, the accuracy of MALDI-TOF-MS reached 100%. The most frequently isolated bloodstream *Candida* species include *C. albicans, C. tropicalis, C. glabrata*, and *C. parapsilosis*, which accounted for 67.9% of our total collection. The correct identification rate of the four subspecies was 100%. The identification accuracy of other subspecies did not reach 100%, and the organisms that were not identified were not in the database library. The limitations of MALDI-TOF-MS are reflected in the number of species in the database. There is a disagreement between those who want to continue adding new species and those who believe that the current database already has sufficient clinical coverage. However, some specific *Candida* species, such as *C. auris*, cannot be identified by Bruker Biotyper and can only be correctly identified when using a library containing *C. auris [*27].

## Conclusion

Our study evaluated the accuracy of MALDI-TOF-MS in the identification of four most frequently isolated bloodstream *Candida* species, which can provide a new means and the gold standard for clinical diagnosis. In summary, MALDI-TOF-MS has proven to be a reliable and rapid method for identification of four most frequently isolated bloodstream *Candida* species. Further research is necessary to determine whether MALDI-TOF-MS can be used as a clinical diagnostic standard for the identification of *Candida*.

## Abbreviations

PICOS: Participant Intervention Comparison Outcome and Study
QUADAS-2: Quality Assessment of Diagnostic Accuracy Studies
RUO: research only
